# Fibroblast Senescence in Idiopathic Pulmonary Fibrosis

**DOI:** 10.3389/fcell.2020.593283

**Published:** 2020-11-25

**Authors:** Yifan Lin, Zhihao Xu

**Affiliations:** Department of Respiratory and Critical Care Medicine, The Fourth Affiliated Hospital, School of Medicine, Zhejiang University, Yiwu, China

**Keywords:** cellular senescence, aging, idiopathic pulmonary fibrosis, fibroblast, senolytics

## Abstract

Aging is an inevitable and complex natural phenomenon due to the increase in age. Cellular senescence means a non-proliferative but viable cellular physiological state. It is the basis of aging, and it exists in the body at any time point. Idiopathic pulmonary fibrosis (IPF) is an interstitial fibrous lung disease with unknown etiology, characterized by irreversible destruction of lung structure and function. Aging is one of the most critical risk factors for IPF, and extensive epidemiological data confirms IPF as an aging-related disease. Senescent fibroblasts in IPF show abnormal activation, telomere shortening, metabolic reprogramming, mitochondrial dysfunction, apoptosis resistance, autophagy deficiency, and senescence-associated secretory phenotypes (SASP). These characteristics of senescent fibroblasts establish a close link between cellular senescence and IPF. The treatment of senescence-related molecules and pathways is continually emerging, and using senolytics eliminating senescent fibroblasts is also actively tried as a new therapy for IPF. In this review, we discuss the roles of aging and cellular senescence in IPF. In particular, we summarize the signaling pathways through which senescent fibroblasts influence the occurrence and development of IPF. On this basis, we further talk about the current treatment ideas, hoping this paper can be used as a helpful reference for future researches.

## Idiopathic Pulmonary Fibrosis is an Aging-Related Disease

Idiopathic pulmonary fibrosis (IPF) is an interstitial lung disease of unknown etiology, characterized by massive deposition of extracellular matrix (ECM) in the lung interstitium, leading to the irreversible and slowly progressive destruction of lung structure and function (Raghu et al., 2011). Most patients with IPF have a median survival of only 2–4 years, and respiratory failure due to IPF progression is the most common cause of intensive care unit (ICU) admission and death for IPF patients (Saydain et al., 2002; Fernández Pérez et al., 2010; Ley et al., 2011). Multiple risk factors increase IPF disease development risk in a single or coordinated manner (Raghu et al., 2011). The endogenous risk factors of IPF include genetic background, aging, gender, and pulmonary microbiology, while the exogenous risk factors include smoking, environmental exposure, and air pollution, especially the dust or organic solvents exposure in the occupational population (Iwai et al., 1994; Baumgartner et al., 2000). Comorbidities such as gastroesophageal reflux, obstructive sleep apnea, and diabetes mellitus are also risk factors (Raghu et al., 2011). Among them, aging is one of the most significant risk factors for IPF.

The risk of interstitial lung disease increases with age. The risk of IPF illness in the elderly over 70 is 6.9 times higher than in the people aged over 40 (Choi et al., 2018). With the advancing age, IPF patients also show more interstitial changes in chest high-resolution computed tomography (HRCT) (Fell et al., 2010). Extensive epidemiological data confirms IPF as an aging-related disease (Hutchinson et al., 2014). The prevalence of IPF is 4.0 per 100,000 in people aged 18–34 years, and this rate is 227.2 per 100,000 in people aged 75 years or older, basing on data recorded in a large American health insurance database from 1996 to 2000 (Raghu et al., 2006). In Japan, from 2003 to 2007, the cumulative prevalence and incidence of IPF are 10.0 and 2.23 per 100,000, respectively, and the incidence also increases with age (Natsuizaka et al., 2014). Further, data from IPF patients in the United Kingdom shows a 35% increase in the incidence of IPF during the study period from 2000 to 2008, with a total incidence of 744 cases per 100,000 population (Navaratnam et al., 2011). From 2004 to 2010, the cumulative annual prevalence of IPF in American adults aged 18–64 increases from 13.4 per 100,000 in 2005 to 18.2 per 100,000 in 2010 (Raghu et al., 2016). Thus, the prevalence and incidence of IPF seem to be on the rise in recent years, partly due to the improved diagnostic methods (Raghu et al., 2014).

Researchers have been trying to figure out how the connections between IPF and aging were established. For one thing, the respiratory system itself shows signs of aging with years. Significant increases in peribronchial collagen and progressive fibrosis are observed in the lungs of natural aging mice (Calabresi et al., 2007). In asymptomatic elders, imaging findings associated with interstitial lung disease are more common, rare in younger populations (Copley et al., 2009). On the other hand, aging is involved in the occurrence and development of IPF disease. Previous studies have identified several common characteristics of aging, and cellular senescence is included as a significant one (Hayflick and Moorhead, 1961; Hayflick, 1965). Aging refers to the decline of various physiological functions and the degeneration of tissues and organs in individuals, which is gradually formed with physiological age growth. Along with the timeline getting closer to the end of the lifespan, the individuals inevitably suffer from this complex natural phenomenon. Quite differently, cellular senescence describes the physiological cell state, which is non-proliferative but living. When the damage to the cells is not extreme enough to initiate the death program but still severe to a certain extent, the damaged cells will begin a senescence program and become senescent (Hayflick and Moorhead, 1961). Aging is based on the accumulation of senescent cells, but cellular senescence can be detected no matter the physical age. Apart from aging, cellular senescence is also involved in a wide range of physiological activities varying from embryonic development, tissue renovates, and wound healing to tumor suppression. Perhaps because of this, the role cellular senescence plays in IPF is more uncertain than aging.

When compared with the age-matched control group, the primary fibroblasts isolated from the lung of IPF patients showed more senescent characteristics, which indicates that cellular senescence is persistent and intense in IPF patients (Álvarez et al., 2017). Under normal circumstances, lung fibroblasts only exist as the mesenchymal cells, located between the epithelial cells in the alveoli or trachea and the endothelial cells in the blood vessels. The alveolar epithelial cells can be harmed by pathogenic microorganisms, dust, drugs, chemicals, oxygen-free radicals, and other factors (Iwai et al., 1994; Baumgartner et al., 2000). Once the alveolar epithelial cells are damaged, while type II alveolar epithelial cells (AEC II) proliferate and differentiate into many flattened type I alveolar epithelial cells (AEC I) to repair the injury, lung fibroblasts are also activated. Activated lung fibroblasts proliferate locally and migrate to the injured area, then they differentiate into myofibroblasts, produce a large number of ECM components, and exhibit contractile function. The accumulated myofibroblasts gradually become senescent after the normal repair progress of lung injury, thus reducing fibroblasts’ activation and limiting the progression of fibrosis. From this point of view, senescent lung fibroblasts play a protective role, for they stopping the deposition of ECM with the end of the repair process (Desmoulière et al., 1995). However, using senolytics removing senescent fibroblasts leads to decreased pulmonary fibrosis in mice model of IPF (Baker et al., 2011; Schafer et al., 2017). What’s more, senolytics effectively improve the lung function in both the IPF mice model and IPF clinical patients, with well tolerance and security (Schafer et al., 2017; Justice et al., 2019). This evidence from the other side indicates the destructive effect of senescent lung fibroblasts, and the decline in the ability of senescent fibroblasts to degrade ECM may be one explanation (Schafer et al., 2017). There are practical reasons for these inconsistent results. In most cases, lung tissues of IPF patients are obtained through percutaneous lung biopsy or pneumonectomy for clinicopathological diagnosis. For ethical reasons, clinicians have to minimize the trauma to patients in invasive procedures or operations, and there are few remaining lung tissues that can be used for scientific researches after meeting diagnostic purposes. Thus in some relevant studies, para-cancer tissue with normal microscopic appearance is used as the control group of IPF lung tissue. It is doubtful whether the concluded results based on those control are actually effective.

## Senescent Fibroblasts in Idiopathic Pulmonary Fibrosis

**FIGURE 1:**
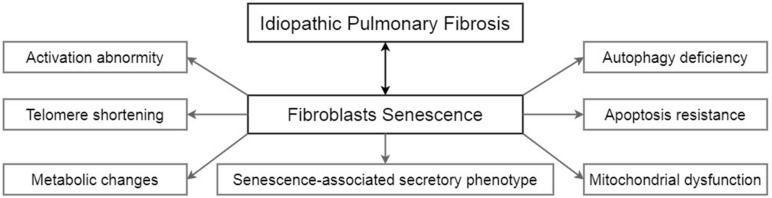
The characteristics of senescent fibroblasts in IPF.

**FIGURE 2:**
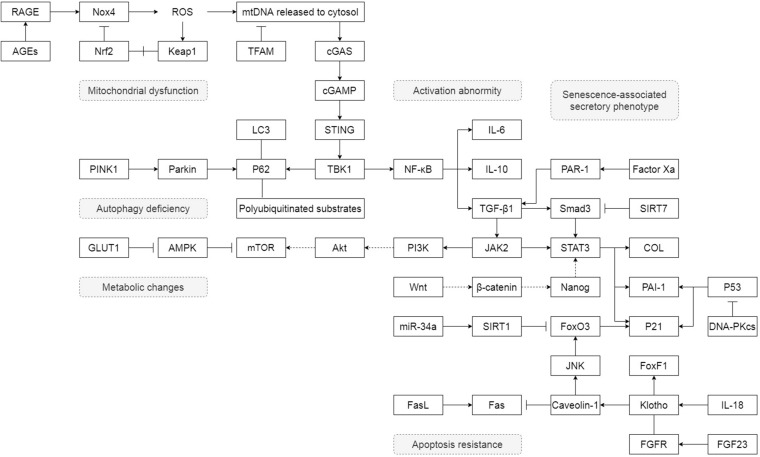
The detail of how senescent fibroblasts affect IPF through the specific molecular signaling pathways. “→” represents “activation”; “⊣” represents “inhibition”; “—” represents “association”; “+” represents “disassociation.”

Unlike lung epithelial cells, which are more like the sensor to external injury and stimulation, pulmonary fibroblasts act as the fibrosis process’s direct executor during the disease. Senescent fibroblasts in IPF are abnormally activated, accompanied by telomere shortening, metabolic reprogramming, mitochondrial dysfunction, apoptosis resistance, insufficient autophagy, and senescence-associated secretory phenotypes (SASP) (Figure 1). Most of these characteristics of IPF senescent fibroblasts, in turn, promote the occurrence and development of IPF. The following part will clearly illustrate how senescent fibroblasts affect IPF through the specific molecular signaling pathways and piece these fragmented results of experiments together to map a signal regulating network in senescent fibroblasts (Figure 2).

### Activation Abnormity

The most fundamental feature of IPF is the deposition of ECM, which results from the abnormal activation of lung fibroblasts (Blokland et al., 2020). The activation of lung fibroblasts involves a series of cell behaviors changes such as proliferation, migration, and ECM production. Primary lung fibroblasts isolated from the lung tissue of IPF patients exhibit a difference in cellular senescence degrees compared with age-matched controls. These fibroblasts increase the levels of senescence-associated β-galactosidase (SA-β-gal), P16, P21, P53, and SASP. The morphology of the IPF lung fibroblasts is enlarged and flat, similar to the morphological changes happening in fibroblasts with replication failure. The doubling time of IPF pulmonary fibroblasts is slow during cell passage *in vitro*, demonstrating the declined proliferation capacity (Ramos et al., 2001; Yanai et al., 2015). These IPF lung fibroblasts also rapidly accumulate the activation markers α-smooth muscle actin (α-SMA) in primary culture, suggesting that these cells are also activated fibroblasts, but may not be equivalent to the senescent myofibroblasts (Yanai et al., 2015; Álvarez et al., 2017).

It is still controversial whether senescent fibroblasts in IPF are equal to senescent myofibroblasts. Some studies suggest that this problem is essentially a difference in experimental design, whether the researchers consider the different expressions of α-SMA protein or other specific identification of myofibroblast phenotype. Nevertheless, more importantly, in head and neck squamous cell carcinoma and esophageal adenocarcinoma, the senescent cancer-related fibroblasts show the same molecular expression, ultrastructure, and contractile properties as the typical TGF-β-induced myofibroblasts. Both cells are α-SMA positive, but RNA sequencing shows that there are significant differences in transcriptome between two kinds of fibroblasts, especially genes related to ECM deposition and tissue remodeling. The senescent fibroblasts with positive α-SMA do not mean that they have fibrogenic properties or they are senescent myofibroblasts. In other words, in IPF, the senescent fibroblasts may only be activated in several limited aspects (Mellone et al., 2016; Blokland et al., 2020). The development of omics research and biological big data analysis gives us the confidence to find this answer in the future.

### Telomere Shortening

When normal human diploid cells are serially cultured *in vitro*, cells will stop proliferation after a limited number of divisions, and this is the earliest description of cellular senescence (Hayflick and Moorhead, 1961; Hayflick, 1965). Subsequent experiments afterward show that this halt in proliferation is induced by telomere shortening. Telomeres are gradually worn down with the increase of passage times of fibroblasts (Harley et al., 1990), and the loss of telomere protection directly exposes chromosome DNA to danger, which may then lead to cellular senescence through DNA damage checkpoint response (d’Adda di Fagagna et al., 2003; McDonough et al., 2018). The relationship between telomere shortening and cellular senescence is always mentioned in IPF. Lung fibroblasts isolated from IPF patients have shorter telomere lengths than age-matched controls (Álvarez et al., 2017), and these cells exhibit accelerated replicative senescence during the primary culture process (Yanai et al., 2015).

Apart from the number of cell divisions, telomere length is also affected by telomerase. Telomerase consists of three parts: telomerase RNA (hTR), telomerase synergistic protein 1 (hTP1), and telomerase reverse transcriptase (hTRT). Telomerase can make catalytic reverse transcription and provide the needed RNA template. Both functions are essential guarantees of normal telomere length. Multiple mutations of hTRT and hTR gene have been found in IPF patients, and they all lead the way to telomere shortening (Armanios et al., 2007; Tsakiri et al., 2007; Alder et al., 2008; Fingerlin et al., 2013). Among them, V144M, R865C, and R865H mutants of hTRT are more significant, and cell experiments determine that V144 and R865 in TRT are two critical residues required for ensuring normal function of cell telomerase (Tsang et al., 2012). However, further studies have shown that hTRT increases the viability of lung fibroblasts, which is beneficial to fibrosis development (Liu et al., 2007, 2013). Differently, hTRT protects AECII from senescence to ameliorate pulmonary fibrosis, suggesting the cell-type-specific role of hTRT in disease (Liu et al., 2019). The performance of telomerase activator GRN510 in external treatment also elucidates the existence of cell-type specificity. GRN510 only shows telomerase activation and cell lifespan prolongation in small airway epithelial cells, but not in lung fibroblasts (Le Saux et al., 2013). However, the shorter telomere length in a significant proportion of IPF patients can’t be explained by the mutant gene encoding telomerase (Cronkhite et al., 2008). This question may be related to the complex mechanism of telomere maintenance. Telomere elongation requires the assistance of telomere elongation helicases, and mutations in the gene RTEL1, an autosomal dominant trait, have also been detected in IPF patients (Kannengiesser et al., 2015). Besides, DNA needs to be bound to the telomere binding protein during telomere formation; thus, the lack of this protein prevents telomere assembly. In AEC II, the deletion of TRF1, a gene that encodes telomere binding protein, successfully constructs a mice model of IPF, showing lung remodeling and pulmonary fibrosis (Povedano et al., 2015). Nevertheless, the absence of TRF1 in collagen-expressing cells only causes pulmonary edema other than fibrosis (Naikawadi et al., 2016). Briefly, telomere shortening and corresponding senescent phenotypes are shown in IPF fibroblasts, but conversely, it seems that fibroblast senescence induced by short telomere does not cause IPF (Yanai et al., 2015; Naikawadi et al., 2016). The association between telomere shortening and cell senescence may merely occur in AEC II, not in pulmonary fibroblasts, showing cell-type specificity (Povedano et al., 2015; Naikawadi et al., 2016). Interestingly, more telomere-associated foci (TAFs) independent of telomere length are detected from lung fibroblasts in IPF mice models than in normal mice (Schafer et al., 2017). TAFs are the oxidative DNA damage located in telomeric G-reach repeats (Hewitt et al., 2012), and their presence suggests that telomeres are still involved in the fibroblast senescence in IPF through another unknown way.

### Metabolic Changes

In primary lung fibroblasts obtained from aged mice, glucose transporter protein 1 (GLUT1) dependent glycolysis is activated. By down-regulating the activity of the AMP-activated protein kinase (AMPK), this glycolytic pathway promotes the protein expression of α-SMA in cells (Cho et al., 2017). And in the studies of human metabolomics, lung tissues of IPF patients also show significant differences in energy metabolism compared with healthy controls, especially the up-regulation of the glycolytic pathway (Zhao et al., 2017). This switch is somewhat similar to the Warburg effect in tumor cells. Warburg effect refers to the behavior that tumor cells tend to metabolize glucose into lactic acid through glycolysis under aerobic conditions, unlike normal cells that generate energy through aerobic oxidation of glucose. With this abnormal glucose metabolism behavior, tumor cells evade the normal apoptosis process and enhance their proliferation and migration ability. Moreover, in IPF, both the aberrant activation of fibroblasts and the substantial synthesis of ECM require enhanced energy generation to meet this biosynthetic requirement (Selvarajah et al., 2019).

The increased glycolysis is also the result of multiple signal transduction and interaction within the senescent fibroblasts. Hypoxia resulted from the progress of IPF disease will lead to the disorder of cell energy metabolism, but on the other hand, hypoxia also up-regulates the expression of hypoxia-inducible factor-1α (HIF-1α) protein and mRNA. HIF-1α mediates the overexpression of pyruvate dehydrogenase kinase 1 (PDK1) gene, which in turn activated glycolysis and increased the activation of lung fibroblasts (Goodwin et al., 2018). Consistent with the experimental results obtained from aged mice, up-regulated TGF-β1 signaling in IPF fibroblasts promotes GLUT1 mRNA expression through the typical Smad2/3 pathway, activates GLUT1-dependent glycolysis, and accelerates cell proliferation and production of fibrogenic mediators (Andrianifahanana et al., 2016). Moreover, at the fibrogenic regions in IPF patients and IPF mice models, fibroblasts associated with metabolically active and apoptosis-resistant show lower AMPK activity (Rangarajan et al., 2018). In addition, another product of glycolysis, lactic acid, is also found in the lung tissues of IPF. Lactate activates latent TGF-β1 by changing the pH value of the internal environment and then promotes the occurrence of fibrosis (Kottmann et al., 2012). Conversely, glycolysis inhibition attenuates both lung fibroblast activation and the growth-promoting phenotype of IPF fibroblasts (Dias et al., 2019). Fructose-1, 6-bisphosphate is an intermediate product of glycolysis, which can decrease the proliferation of fibroblasts and the cell’s ability to produce ECM, thus halting the occurrence of pulmonary fibrosis (Xie et al., 2015). Increased glycolysis is a consequence of cellular senescence, but in another way, it also promotes pulmonary fibrosis. This may explain the inhibition effect of pulmonary fibrosis by AMPK activators such as metformin (Rangarajan et al., 2018; Kheirollahi et al., 2019).

Apart from glycolysis, metabolic heterogeneity in IPF fibroblasts also includes changes in bile acid, heme, amino acid, and lipid metabolism (Zhao et al., 2017). TGF-β1 increases the production of activated transcription factor 4 (ATF4) in fibroblasts. ATF4 is the central transcriptional regulator of amino acid metabolism and provides glucose-derived glycine to meet the amino acid requirements of cells associated with enhanced collagen production (Selvarajah et al., 2019). There is also a metabolic process related to glutamine, a critical metabolic process in which glutaminase converts glutamine into glutamic acid and then into the TCA cycle metabolite α-ketoglutarate (α-KG), mediating the resistance to apoptosis of fibroblasts (Bai et al., 2019). Also, advanced glycation end products (AGEs), as products of non-enzymatic reactions of fats and proteins with various oxidants during the senescent process, are a group of stable covalent compounds. By acting on the receptor of AGEs (RAGEs), AGEs also affect fibroblast activation (Machahua et al., 2016).

### Mitochondrial Dysfunction

The mitochondrial dysfunction triggers include transmembrane potential loss in the mitochondrial inner membrane, down-regulation of electron transport chain (ETC) function, and reduction of key metabolites entering mitochondria from the cytoplasm (Nicolson, 2014). These alterations reduce mitochondrial oxidative phosphorylation and reduce ATP production (Nicolson, 2014). They then switch on several age-related changes, particularly the overproduction of pro-inflammatory and pro-oxidative signals (Correia-Melo et al., 2016). An overall decrease in mitochondrial mass and function are observed in IPF lung fibroblasts than healthy controls (Álvarez et al., 2017; Caporarello et al., 2019). The declining mitochondrial mass is associated with an abnormality in mitochondrial biogenesis and mitophagy. The decreased mitochondrial function is manifested as increased reactive oxygen species (ROS) production, decreased mitochondrial membrane potential, and mitochondrial respiratory chain complex in senescent fibroblasts (Luo et al., 2013). They both ultimately drive the senescence phenotypes of the cells and promote fibrosis progression (Wiley et al., 2016).

Mitophagy, which occurs in normal cells to maintain mitochondrial homeostasis, is down-regulated in senescent fibroblasts, leading to decreased PTEN-induced putative kinase 1 (PINK1) (Sosulski et al., 2015). Inhibited mitophagy activates the downstream pathway of platelet-derived growth factor receptor (PDGFR), further amplifying the mitophagy process (Kobayashi et al., 2016). Besides, levels of peroxisome proliferator-activated receptor γ coactivator-1α (PGC1α) and mitochondrial transcription factor A (TFAM) are steadily inhibited in IPF cells (Hecker et al., 2014; Bernard et al., 2015, 2017; Caporarello et al., 2019). Furthermore, dysfunctional mitochondria produce large amounts of ROS, including peroxides, superoxides, and hydroxyl radicals. ROS produced by lung fibroblasts rapidly and sensitively promotes cell proliferation and activation in a dose-dependent manner, and ROS is also endogenous damage to lung epithelial cells (Murrell et al., 1990; Waghray et al., 2005). The production of intracellular ROS is related to the decrease of mitochondrial membrane potential, which could be restored by the deactivation of the mammalian target of rapamycin (mTOR). mTOR activity also affects the signal transduction between mitochondria and nucleus, and the coordinated expression of mitochondrial and nuclear genes is a necessary condition for maintaining the normal function of mitochondria (Lerner et al., 2013). Reactive oxygen-producing enzyme NADPH oxidase 4 (Nox4) is another source of ROS (Jain et al., 2013; Bernard et al., 2017). NOX4 regulates the protein abundance of α-SMA and collagen by controlling the activation of Smad2/3 and regulated PDGF-induced fibroblast migration (Amara et al., 2010). What is worse, the antioxidant capacity of the nuclear factor E2-related factor 2 (Nrf2) is impaired in senescent fibroblasts, resulting in the Nox4-Nrf2 redox imbalance, which ultimately promotes the activation of fibroblasts and the formation of senescence phenotypes (Hecker et al., 2014; Bernard et al., 2015, 2017).

In addition, signal transduction and transcriptional activator factor 3 (STAT3) is necessary for the ETC of mitochondrial complexes I and II to remain activation and is involved in coordinating intracellular homeostasis (Wegrzyn et al., 2009). The elevation of superoxide concentration in senescent lung fibroblasts continuously promotes the translocation of STAT3 to the nucleus and mitochondria, mediates the metabolic function of mitochondria, and raises the nuclear transcription level of senescence phenotypic related genes (Gough et al., 2009; Waters et al., 2019). Besides, in IPF lung fibroblasts, the promotion effect on cell activation by TGF-β1 signaling is accompanied by a decrease in the expression of silent mating type information regulation2 homolog-3 (SIRT3) gene (Sosulski et al., 2017). SIRT3 plays a deacetylase role in mitochondria to regulate mitochondrial health (Bindu et al., 2017) and plays a key role in repairing mitochondrial DNA damage and protecting mitochondrial integrity, too (Cheng et al., 2013). The depletion of endogenous SIRT3 will increase ROS production and mitochondrial DNA (mtDNA) damage, leading to the progression of pulmonary fibrosis (Sundaresan et al., 2015; Bindu et al., 2017). Damaged mtDNA, which should not be present in the cytoplasm, is released into the cytoplasm and is sensed by the cyclic GMP-AMP synthase (cGAS). cGAS catalyzes the production of cyclic guanosine acid (cGAMP), which activates STING protein and promotes the senescence of lung fibroblasts (Schuliga et al., 2020). What is more, superoxide enhances DNA damage response (DDR), which also amplifies age-related effects in lung fibroblasts as part of a positive feedback process (Schuliga et al., 2018).

### Apoptosis Resistance

Apoptosis is an autonomously ordered death controlled by genes and can maintain homeostasis. In the restoration process of lung injury, the activated fibroblasts show changes corresponding to apoptosis and gradually disappear at the end (Desmoulière et al., 1995). Apoptosis of IPF lung fibroblasts is reduced compared with age-matched controls. However, in IPF, apoptosis’s stimulation has a higher frequency and level, so the decreased sensitivity of cells to apoptotic signals is a convincing explanation (Álvarez et al., 2017). Apoptosis resistance of senescent fibroblasts is associated with the pathogenesis of IPF (Cha et al., 2010).

Reduced pro-apoptotic proteins Bak and Bax and increased anti-apoptotic protein Bcl-2 family proteins are found in IPF senescent fibroblasts (Moodley et al., 2003; Sanders et al., 2013). The accumulation of Bcl-2 family proteins, which include Bcl-2, Bcl-W, and Bcl-XL, contributes to apoptotic stimuli resistance in senescent cells (Ricci et al., 2013a; Yosef et al., 2016). Alterations in the expression of pro-apoptotic and anti-apoptotic genes are associated with histone modification and DNA methylation (Sanders et al., 2014). In contrast to the Bax gene, the acetylation of histone H4K16 (H4K16Ac) is significantly enriched in the Bcl-2 gene while the trimethylation of histone H4K20 (H4K20Me3) is significantly decreased (Sanders et al., 2013). These site-specific histone modifications regulate the expression of the Bcl-2: Bax gene in senescent fibroblasts, leading to the anti-apoptotic phenotypes (Sanders et al., 2013). Intracellular TGF-β1 signaling increases the Bcl-2 protein level by activating JAK2 and STAT3 (Milara et al., 2018). Both the levels of Bcl-2 and Bax protein in fibroblasts are STAT3-dependent (Moodley et al., 2003), and inhibition of STAT3 signaling can block this resistance to apoptosis (Pechkovsky et al., 2012). Further, the mechanical sensitivity signal transduction pathway up-regulates the expression of the Bcl-2 gene in activated fibroblasts through activation of the Rho/ROCK pathway, reducing fibroblast apoptosis reduction, and contributes to the continuous fibrosis process (Zhou et al., 2013). Cytokines IL-6 and IL-11 increase the expression of Bcl-2 mRNA in IPF lung fibroblasts to inhibit apoptosis (Moodley et al., 2003). The plasminogen activator inhibitor 1 (PAI-1) level in mouse lung fibroblasts increases significantly with age, accompanied by a decrease in apoptosis in fibroblasts (Huang et al., 2015; Marudamuthu et al., 2015).

Also, senescent IPF lung fibroblasts are found to be highly resistant to Fas ligand-induced (FasL) and TNF-associated apoptotic ligand-induced (TRAIL) apoptosis. FasL, TRAIL, and Caveolin-1 (Cav-1) protein abundance is decreased, and AKT activity is increased in these cells (Hohmann et al., 2019). TGF-β1 mediates the down-regulation of Cav-1 in fibroblasts through the MAPK signaling pathway (Sanders et al., 2015), and Cav-1 loss gives fibroblasts anti-apoptotic properties (Shivshankar et al., 2012). The eukaryotic elongation factor 2 kinase (eEF2K) interacts with the MAPK signaling pathway to activate the apoptosis of lung fibroblasts (Wang Y. et al., 2018). Increased AKT activity also appears in a variety of signaling pathways involved in apoptosis resistance. Activation of the PI3K/AKT/mTOR pathway helps IPF lung fibroblasts resist apoptosis (Romero et al., 2016). The low activity of PTEN leads to the inactivation of the transcription activator FoxO3a through the PTEN/Akt-dependent pathway, down-regulates the expression of Cav-1 and Fas gene, and thus gives IPF fibroblasts an obvious anti-apoptotic phenotype (Nho et al., 2013). In addition, activated AKT results in the enhancing decoy receptor-3 (DcR3) level in IPF fibroblasts. Fas is a death receptor in the tumor necrosis factor receptor superfamily that binds to FasL to induce apoptosis (Cha et al., 2010), and DcR3 is another tumor necrosis factor receptor that competitively binds to FasL to protect IPF fibroblasts from FasL-induced apoptosis (Im et al., 2016). P53 is also involved in regulating apoptosis and thus affect the survival advantage of IPF fibroblasts. The p53 protein level increases in senescent fibroblasts and decreases after apoptosis is induced by an inhibitor of anti-apoptotic proteins (Yosef et al., 2016). P53 is responsible for transcription and activation of apoptosis-related genes, such as PUMA (also known as BCL-2-binding component 3) and Bax (He et al., 2013). Human 8-oxoguanine DNA Glycosylase (hOGG1) protects cells from apoptosis induced by oxidative stress ahead of the p53 dependent pathway (Youn et al., 2007). On the other hand, the protein expression of homeodomain interacting protein kinase 2 (HIPK2) is low in fibroblasts from IPF patients, and its interaction with p53 weakens the cells’ ability to make decisions between cell cycle withdrawal and apoptosis. Restoring the HIPK2 protein level in IPF cells reduces the chemoresistance (Ricci et al., 2013b). In senescent fibroblasts, the post-translational modification of p53 makes p53 preferentially occupy the promoters of growth inhibition genes, rather than apoptosis regulators (Jackson and Pereira-Smith, 2006). Further studies are still needed to elucidate the role of p53 protein in apoptosis resistance of senescent cells.

### Autophagy Deficiency

Autophagy is responsible for the retrieval of various components by degrading organelles and macromolecules to provide the raw material for reconstructing cellular structures, carefully maintaining the usual fate of fibroblasts (Sosulski et al., 2015). The cystic autophagosome is formed during the autophagy process. Meanwhile, p62 mediates the binding between the ubiquitinated protein and microtubule-associated protein light chain 3 (LC3), participating in autophagosome formation (Patel et al., 2012). Generally, autophagy is non-selective, but in pathological conditions, autophagy can act as a selective barrier to encapsulate certain intracellular abnormal components for isolation (Sosulski et al., 2015). Mitophagy, as a part of selective autophagy, has been discussed in mitochondrial dysfunction. Autophagy plays an essential regulatory role in cellular senescence, and autophagy deficiency is closely related to IPF fibroblast senescence (Araya et al., 2013). Autophagy is reduced in elderly mice after lung injury, accompanied by corresponding increases in oxidative protein and lipofuscin levels. Lung fibroblasts activated by TGF-β1 are characterized by reduced autophagy flux (Sosulski et al., 2015).

Autophagy-related biomarkers in lung fibroblasts consist of apoptotic effector protein Beclin1, LC3, p62, and the autophagosome number (Patel et al., 2012; Xu et al., 2019). All of them imply decreased autophagy activity in lung tissues of IPF patients (Patel et al., 2012). Beclin1, a key regulator of autophagy in IPF lung fibroblasts, is down-regulated compared with normal lung fibroblasts (Patel et al., 2012; Ricci et al., 2013a). IL-37 can enhance the beclin1-dependent autophagy pathway in IPF fibroblasts (Kim et al., 2019). Cellular senescence is conducive to mediating mTOR-related pathways in lung fibroblasts to reduce autophagy as an adaptive response to stress (Patel et al., 2012; Romero et al., 2016). Downstream molecules of mTORC1 include eEF2K and TFEB (Hait et al., 2006; Wang K. et al., 2018). eEF2K can phosphorylate and inactivate eEF2 and regulated the autophagy activity of lung fibroblasts and IPF development through the MAPK signaling pathway (Wang Y. et al., 2018). Inhibition of mTOR activation can stimulate autophagy, which is characterized by increased beclin1 and LC3 levels as well as autophagosome formation (Chitra et al., 2015).

Autophagy is also involved in the regulation of ECM formation. Studies have shown that increasing the autophagy clearance rate to type 1 collagen by lung fibroblasts could reduce the aggressiveness of IPF fibroblasts (Surolia et al., 2019). In IPF, TGF-β1 can induce the overproduction of ECM components such as collagen and fibronectin in lung fibroblasts. Although TGF-β1 also induces LC3B accumulation in parallel, this autophagy marker’s content is significantly decreased in IPF lung fibroblasts (Ghavami et al., 2018). Changes in the PTEN-Akt-mTOR axis make IPF fibroblasts to maintain collagen overproduction’s pathological phenotype by inhibiting autophagy. Decreased expression of Akt gene direct target FoxO3a inhibits the production of autophagy marker LC3B on the collagen matrix, thereby inhibiting the autophagy response of IPF fibroblasts to collagen (Nho and Hergert, 2014; Im et al., 2015).

### Senescence-Associated Secretory Phenotype

Senescent cells typically secrete a complex set of factors known as senescence-associated secretory phenotype (SASP), a unique cellular senescence feature. Elevated transcription levels of these factors can be synchronously detected in the cells (Schafer et al., 2017; Álvarez et al., 2017). SASP in IPF senescent fibroblasts includes proinflammatory cytokines (such as TNF-α, TGF-β, IL-1β, IL-6, IL-8, IL-10, IL-18), chemokines (such as CXCL1, MCP-1), growth regulators (such as FGF, CTGF, GM-CSF, M-CSF, PDGF), matrix metalloproteinases (such as MMP-2, MMP-3, MMP-9, MMP-10, MMP-12), and leukotrienes (LTs, such as LTA4, LTB4, LTC4, LTD4) (Kortlever et al., 2006; Acosta et al., 2008; Rodier et al., 2009; Kojima et al., 2012; Aoshiba et al., 2013; Demaria et al., 2014; Le et al., 2014; Hayakawa et al., 2015; Jun and Lau, 2017; Schafer et al., 2017; Álvarez et al., 2017; Wiley et al., 2019; Zhang et al., 2019; Blokland et al., 2020). Thus, SASP is essentially a concept that belongs to secretomes, which cannot be fully described by several biomarkers’ up-regulation or down-regulation. The composition of SASP changes dynamically over time; for example, Notch1 activity controls the transition of secretory components from TGF-β-dependent to inflammatory factor-dependent (Hoare et al., 2016). SASP turns on the switch of cell senescence, promotes the senescence of cell itself through autocrine, and propagates senescence through paracrine.

The SASP activated in senescent cells is a self-amplified secretory network (Acosta et al., 2008). In IPF senescence fibroblasts, mitochondrial dysfunction decreases the NAD+/NADH ratio, while NAD+ metabolism controls the pro-inflammatory SASP production independently of senescence-related growth stagnation (Correia-Melo et al., 2016; Wiley et al., 2016; Nacarelli et al., 2019). IL-1 and TGF-β can induce Nox4 mRNA expression, suggesting a mechanism between SASP and DNA damage signals (Hubackova et al., 2012). In IPF, increased secretion of SASP usually occurs only after persistent DNA damage signals associated with senescence rather than transient DNA damage response (DDR) (Rodier et al., 2009). DDR signal is necessary but not sufficient for the secretion of SASP. The MAPK pathway, activated by multiple stimuli, induces SASP production by increasing the transcriptional activity of NF-κB, which is independent of DDR (Chien et al., 2011; Freund et al., 2011; Aoshiba et al., 2013; Ferrand et al., 2015). In addition, connective tissue growth factor (CTGF) induces fibroblast senescence by mediating ROS accumulation, leading to the activation of p53 and the induction of p16INK4a (Jun and Lau, 2017). Although the secretion of SASP is regulated by the cell cycle arrest associated with the p53 and Rb pathways, inhibition of either p53 or the Rb pathway is not enough to prevent SASP-induced effects (Freund et al., 2011; Ferrand et al., 2015). Both JAK inhibitors and mTOR inhibitors inhibit SASP in fibroblasts (Herranz et al., 2015; Xu et al., 2015). Besides, IL-18 promotes lung fibroblast senescence and the role of SASP in IPF by blocking the Klotho pathway (Zhang et al., 2019). IL-6 induces normal fibroblast senescence by establishing a senescence induction circuit involving STAT3 and insulin-like growth factor-binding protein 5 (IGFBP5) (Kojima et al., 2012). Inhibition of IL-6 reduces pulmonary fibrosis in mice (Le et al., 2014).

Senescent fibroblasts also influence the local microenvironment and the senescence of adjacent cells through SASP in a paracrine manner and finally aggravate IPF disease (Acosta et al., 2013; Blokland et al., 2020). When the conditioned medium obtained from senescent fibroblasts is co-cultured with alveolar epithelial cells, a higher percent of alveolar epithelial cells are blocked at the G2/M stage than the control group co-cultured with blank medium, resulting in reduced proliferation but increased migration of alveolar epithelial cells (Blokland et al., 2020). Increased senescence of bone marrow mesenchymal stem cells (B-Mscs) as an extrapulmonary manifestation of IPF patients is also associated with this. And senescent B-Mscs in IPF is capable of inducing the senescence of normal senescent lung fibroblasts through their paracrine effects (Cárdenes et al., 2018).

## Targeting Senescent Fibroblasts in Idiopathic Pulmonary Fibrosis

**FIGURE 3:**
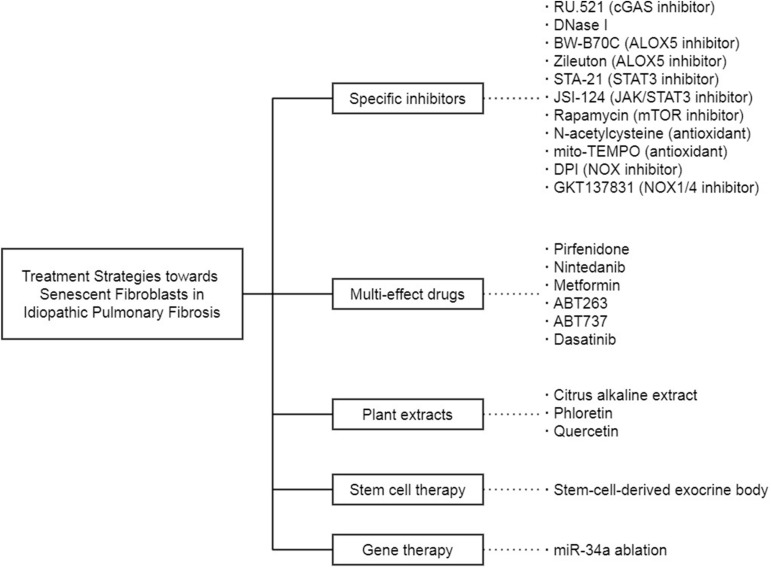
The treatment strategies toward senescent fibroblasts in IPF.

Fibroblast senescence is an important therapeutic target of IPF (Figure 3). In the previous studies, a large part of the treatment strategies toward IPF is proposed on the basis of the premise that specific therapies targeting one selected senescence-related molecule or pathway in senescent fibroblasts can reduce the progression of IPF, and there are some experiments indeed confirm the feasibility of this idea. The selective cGAS inhibitor RU.521 eliminates the senescence of IPF fibroblasts induced by ectopic DNA in the cytoplasm (Schuliga et al., 2020). DNase I, which removes the DNA released into the cytoplasm by abnormal pathways, leads to effects similar to RU.521. 5-lipoxygenase (ALOX5) inhibitors BW-B70C and Zileuton block the synthesis of LTs, which are proved as part of the SASP. Inhibition of ALOX5 activity modulates the proinflammatory and profibrotic effect of LTs on senescent IPF fibroblasts (Wiley et al., 2019). In addition, STA-21, a specific inhibitor of STAT3, reduces the stress-induced senescence of lung fibroblasts (Waters et al., 2019). Furthermore, JSI-124, a dual inhibitor of JAK2/STAT3 more effective than a single inhibitor, inhibits the migration, intracellular autophagy, and senescence of lung fibroblasts (Milara et al., 2018). What is more, mTOR inhibitor Rapamycin, antioxidant N-acetylcysteine (NAC), and mitochondrial-targeted superoxide dismutase mito-TEMPO act on the ROS production and DDR response in mitochondria to suppress IPF fibroblast senescence (Schuliga et al., 2018). The non-selective NOX inhibitor diphenyleneiodonium chloride (DPI) and the specific NOX1/4 inhibitor GKT137831 also attenuate the senescence phenotype of lung fibroblasts (Thannickal and Fanburg, 1995; Hecker et al., 2009, 2014).

Given that fibroblast senescence in IPF cannot be entirely attributed to a single cause, these targeted drugs are probably incapable of achieving satisfactory results due to their limited effectiveness. Concomitant administration of several drugs is likely to fall into the dilemma between the chaos caused by drug interactions and the inability to cover every discovered signal pathways. Thus, researchers turn to drugs with multiple effects. Pirfenidone is a pleiotropic molecule approved to be one of the antifibrotic drugs to IPF. It inhibits the MUC1 phosphorylation and β-catenin activation induced by TGF-β1 and prevents SMAD binding elements from forming or activating, thereby combating the fibroblast senescence in IPF (Ballester et al., 2020). Nintedanib inhibits TGF-β1 signal transduction intracellularly and induces IPF fibroblasts to autophagy, thereby down-regulating ECM production (Rangarajan et al., 2016). Metformin, a first-line antidiabetic drug, regulated the intracellular metabolic pathways, inhibits the TGF-β1 activated collagen formation, and accelerates the reversal of established fibrosis in an AMPK-dependent manner, thereby acts as an effective antifibrosis agent in the lung (Rangarajan et al., 2018; Kheirollahi et al., 2019). The anti-cancer drugs ABT263 and ABT737 can alleviate the senescence of lung fibroblasts by blocking the up-regulation of Bcl-2 family anti-apoptotic proteins, including Bcl-2, Bcl-XL, and Bcl-W (Chang et al., 2016; Yosef et al., 2016; Zhu et al., 2016). Besides, active substances isolated from edible plants are also research objects. Citrus alkaline extracts (Feng et al., 2019) and phloretin (Cho et al., 2017) affect senescent fibroblasts by activating cyclooxygenase-2 (COX-2) and inhibiting GLUT1, respectively. Quercetin reverses the apoptosis resistance in IPF fibroblasts by promoting the transcription of FasL receptor and Cav-1 gene (Hohmann et al., 2019), while the combination of dasatinib and quercetin (D+Q) selectively kills senescent fibroblasts to ameliorate the progression of pulmonary fibrosis in mice (Schafer et al., 2017). What is even more exciting is that the D+Q performs well in a small-scale, open-labeled pilot clinical trial (Justice et al., 2019). The fourteen stable IPF patients selected for this trial are well tolerated to drugs, and their physical function is significantly and clinically meaningfully improved. Although the pulmonary function is unchanged in this pilot study, the physical elevation achieved by D+Q in IPF patients is still worthwhile in the face of the latest anti-fibrosis drugs, Pirfenidone and Nintedanib.

In addition to these drugs mentioned above, there are also other treatments for senescent fibroblasts in IPF. Stem cell therapy is one of them. The exocrine body derived from human amniotic epithelial cells contains many soluble factors that can regulate inflammation and fibrosis pathways and is considered a potential treatment for IPF. These isolated stem-cell-derived extracellular vesicles reduce lung inflammation and fibrosis in mice by nasal drip (Tan et al., 2018). Gene therapy targeting non-coding RNA is also a new train of thought. A non-coding RNA sequence can link with multiple protein-coding RNA sequences complementarily, thus achieving precise transcriptional regulation as a therapy. For example, miRNA-34a is upregulated in lung fibroblasts from elderly mice (Cui et al., 2017a). Furthermore, in the fibrotic lung of miRNA-34a-deficient mice, the senescence phenotype of primary lung fibroblasts is reduced, and the anti-apoptosis ability is enhanced (Cui et al., 2017b). However, it is not easy to guarantee the safety and convenience of gene therapy targeting non-coding RNA in the human body, and there is still a long way to go from a theoretical proposal to clinical practice.

## Conclusion and Perspectives

Cellular senescence is characterized by cell cycle arrest, macromolecular damage, metabolic disorders, and SASP, which are important aging pathways. IPF is an aging-related interstitial lung disease of unknown etiology. The pathogenesis of IPF itself is difficult to be directly and clearly summarized with several abnormalities. Otherwise, it cannot be called “idiopathic.” Although the source of the disease is the key to solve the problem, for IPF patients, intervention in the process of their onset or progression may be a better choice, at least at this stage. We must be clear about the role of cellular senescence in IPF. The related experiments in IPF patients have the problem that the control group is not suitable, while the IPF mice model induced by bleomycin also has the problem of heterogeneity and self-remission. There is no better way to solve the experimental control group’s problems, but the improvement in animal model construction can provide more opportunities for senescence-related research in IPF.

Just as cellular senescence is implicitly multifaceted in IPF, how senescent fibroblasts function is also multifaceted. Senescent fibroblasts exhibit abnormal activation, telomere shortening, metabolic reprogramming, mitochondrial dysfunction, apoptosis resistance, autophagy deficiency, and SASP secretion, involving a variety of molecular signaling pathways. However, we have not yet found a sufficiently unique and universal biomarker for cellular senescence. The signaling pathways in senescent fibroblasts are like a dense web, which tightly controls the IPF disease’s severity. It may not be enough only to untie a knot on the web.

Under these conditions, the idea of using drugs that precisely clear senescent cells are gaining ground. In the selection of senolytics, compounds from nature undoubtedly perform better for their pleiotropic effects as well as security. When we attempt to discover new senolytics from a vast array of natural and human-made compounds, it is also a beneficial idea in our opinion to find appropriate answers from drugs already on the market or in clinical trials. As the old saying goes, the best way to discover a new drug is to start with an old one. Do drugs that can act on multiple senescence-related pathways at the same time also hide anti-aging properties? In the future, more cell experiments and animal experiments will give us the answer. In addition, just as new pathways are constantly confirmed in IPF, the signaling network in senescent fibroblasts we map will also be enriched. We believe that cellular senescence and senolytics will become new lights in the treatment of IPF.

## Author Contributions

Both authors read and approved the manuscript for publication.

## Conflict of Interest

The authors declare that the research was conducted in the absence of any commercial or financial relationships that could be construed as a potential conflict of interest.
